# Ultra‐Conserved Poison Exons Enable Rapid and Safe Splicing Factor Gene Expression Switches: A Hypothesis

**DOI:** 10.1002/bies.70081

**Published:** 2025-11-02

**Authors:** Caroline Dalgliesh, Farimah Ghorbani, Adam J. M. Wollman, David J. Elliott

**Affiliations:** ^1^ Newcastle University Biosciences Institute Newcastle University Newcastle upon Tyne UK

## Abstract

Most vertebrate genes are split up into exons and introns, with exons being spliced together to make mRNA. Many of the proteins involved in splicing, called splicing factors, exert concentration‐dependent effects on gene expression through post‐transcriptional modification of mRNAs. These include the serine/arginine‐enriched (SR) proteins that have essential roles in normal development and physiology. All SR proteins (and many other splicing factors) regulate their own expression levels, often using negative feedback pathways involving alternative splicing of “poison exons” (PEs), which lead to mRNA degradation. The PEs within SR protein genes are encoded by ultra‐conserved genome sequences, suggesting they have been under extreme selective pressure despite not encoding protein sequences. Here, we discuss the hypothesis that PEs enable rapid switches in SR protein concentrations, yet prevent these splicing regulators from increasing to toxic levels that cause cell death or interfere with cell function. This hypothesis is based on analysis of an ultra‐conserved PE in the *TRA2B* gene during male meiosis. Distinct roles for this *TRA2B* PE in different tissues further predict cell type‐specific effects on development and physiology that will need to be experimentally detected using animal models.

## Graphical abstract

Here, we suggest that negative autoregulation (NAR) by poison exon splicing enables splicing factors to rapidly and efficiently switch expression in response to transcription changes. This would prevent potentially dangerous levels of splicing factor proteins accumulating in vulnerable cell types and help explain poison exon ultra‐conservation.

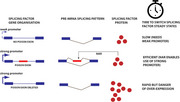

## 1 Introduction

Proper control of gene expression plays a key role in development. Most vertebrate genes are transcribed as precursor mRNAs (pre‐mRNAs) containing exons and introns. This split organisation requires exons to be recognised and spliced together efficiently by the spliceosome, a multi‐subunit complex containing proteins (called splicing factors) and snRNAs [1]. Pioneer splicing factors, including members of the serine‐arginine enriched (SR) protein family, bind to pre‐mRNAs to help the spliceosome recognise exons through a process called exon definition [2, 3]. Some exons are alternatively spliced, being spliced into some mRNAs and left out of other mRNAs. Alternative splicing patterns make a key contribution to normal development and physiology [4, 5, 6]. There are only around 20 000 protein‐coding genes in the human genome, but alternative splicing allows this finite number of genes to encode an expanded number of mRNA isoforms and proteins, sometimes with different functions.

This review discusses the hypothesis that a special type of alternative exon called a poison exon (PE) enables some splicing factors to modulate their expression in a rapid and safe manner during development. PEs are not restricted to splicing factor genes: up to a third of human genes contain PEs [7]. PE inclusion introduces premature translation termination codons, producing unstable mRNAs which are rapidly degraded by a pathway called nonsense‐mediated decay (NMD) [7, 8, 9]. Alternative splicing of PEs coupled to NMD provides a method for controlling gene expression levels post‐transcriptionally [10]. mRNA transcripts containing PEs are rapidly degraded and so often not detected using normal mRNAseq protocols, even if they are initially produced at high levels by transcription. These unstable transcripts, therefore, equate to a “dark matter” of the transcriptome that might have a high throughput but only a transient existence.

**FIGURE 1:**
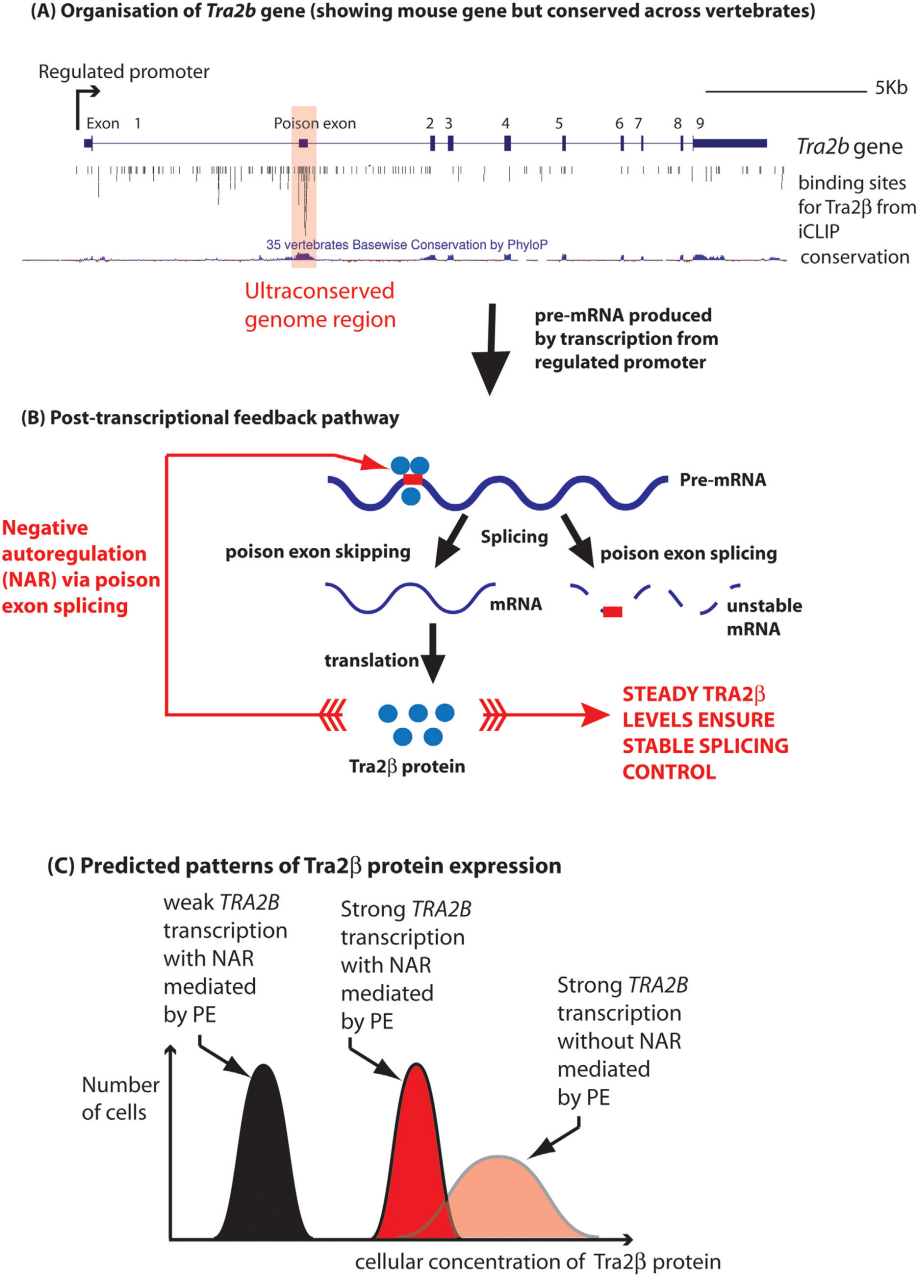
Hypothesis: NAR by PE splicing will enable levels of splicing factors like Tra2β to rapidly and uniformly respond to transcriptional input and maintain cell function/viability. (A) An ultra‐conserved PE is located within the *TRA2B* gene that encodes the splicing factor Tra2β. UCSC screenshot showing the mouse *Tra2b* gene, the position of its promoter element (mapped from the position of its associated CpG island), patterns of Tra2β protein binding identified by iCLIP in the mouse testis, and levels of genome conservation (as a phyloP plot, where bars above the line represent conservation). The *TRA2B* PE has a lod score of 2908 (a measure of conservation level, with a higher number indicating a higher level of conservation) compared to the downstream intron sequence (lod score = 27), and immediately downstream protein coding exon 2 (lod score = 1274). (B) Expression of *TRA2B* produces an mRNA that is translated into Tra2β protein. Once it reaches an appropriate concentration, Tra2β is able to activate splicing of a PE within its own pre‐mRNA. (C) The advantage of using NAR is that it will enable a strongly regulated promoter to establish rapid changes in splicing factor gene expression that are homogenous across the population of cells. However, deletion of the PE would cause strong transcriptional induction of the splicing factor gene without any correction, and potentially lead to a wider range of expression values, and toxic expression levels of the splicing factor.

Our hypothesis that splicing factor PEs enable rapid and safe expression changes of their associated genes is based on a prototypic PE located within the *TRA2B* gene that encodes the splicing factor Tra2β (Figure 1A). The PE of *TRA2B* and the PEs of many other splicing factors are found within ultra‐conserved regions of the genome, indicative of functional importance that has been preserved under high selective pressure. Ultra‐conserved elements were originally defined as regions ≥200 bp in length sharing complete nucleotide identity between the human, mouse, and rat genomes [11], although conservation usually extends between even more divergent species [12]. Cummins et al. recently proposed an updated definition of ultra‐conserved elements as sequences ≥ 100 bp and ≥97% sequence identity in ≥50% of placental mammalian genomes [13].

Many splicing factor genes, including *TRA2B*, use alternatively spliced PEs to negatively autoregulate (NAR) their own expression levels (Figure 1B) [14, 15]. The encoded splicing factor binds directly to the PE within its own pre‐mRNA and activates its splicing, destabilising the resulting mRNA. This equilibrates splicing factor expression: any increase in splicing factor expression will cause more PE splicing, and vice versa. In this way, PE feedback pathways are viewed as homeostatic feedback mechanisms that maintain stable expression levels of splicing factors [16].

Underpinning the importance of maintaining stable concentrations of splicing factors, some diseases, including myotonic dystrophy and amyotrophic lateral sclerosis (ALS), are caused by changes in the nuclear concentration of splicing factors. These changes alter RNA splicing patterns and protein isoforms, leading to disease symptoms [17, 18]. In the neurological disease ALS, high levels of the splicing factor TDP43 accumulate in nuclear inclusions. This lowers the concentration of TDP43 “available” for regulating splicing, causing the accumulation of cryptic splice variants within important neurological genes [19]. Interestingly, expression levels of the *TARDP* gene that encodes TDP43 are controlled by an NMD‐based mechanism (although not via PE splicing), showing these pathways are important [20]. PEs have been directly implicated in human disease. Disease‐causing variant sequences within the PE of the human *SNRPB* gene (that encodes a splicing factor) lead to increased PE inclusion, thereby reducing *SNRPB* mRNA expression and causing cerebro‐costo‐mandibular syndrome [21]. PE splicing control may also be important in development. Splicing inclusion of some PEs within splicing regulator genes is dynamically modulated during organoid development along different lineages [22] and in different tissues [22, 23, 24]. This dynamic regulation may allow cells to maintain the correct balance of splicing factors required at each stage [25]. Targeted deletion of a PE within the gene encoding the spliceosome protein SMNDC1 caused growth defects in both mice and plants [25].

## 2 Hypothesis: Negative Autoregulation is Used to Enable Rapid, Stable, and Safe Changes in Splicing Factor Gene Expression

In this article, we propose that negative autoregulation (NAR) by PE splicing enables splicing factor genes to rapidly and efficiently modulate their expression levels in response to transcriptional cues and generate uniform expression patterns across individual cells. Thus, rather than simply maintaining homeostasis, PEs become particularly important when gene expression patterns are changing.

The hypothesis that splicing factors use PEs to facilitate changes in their gene expression is conceptually similar to the NAR pathways that frequently control the expression of transcription repressor proteins in bacteria. Many bacterial transcriptional repressors negatively autoregulate transcription from their own genes, and this has been investigated at both theoretical and experimental levels [26]. In bacteria, increases in promoter activity can rapidly increase gene expression levels and transcription repressor protein production. NAR cuts off further expression increases when a threshold concentration is reached at which the promoter is efficiently negatively autoregulated by the repressor protein encoded by the gene. In contrast, without NAR, such increased production would necessarily be less efficient, since any promoter activity increase would need to be slower and more controlled to prevent transcription repressor protein over‐production. In bacteria, the combination of strong promoters for the genes encoding transcription repressors coupled to NAR allows gene expression systems to reach an increased steady state of protein production 50% quicker than using a weaker promoter without negative autoregulation [26]. Thus, although counterintuitive, using NAR facilitates rapid dynamic increases in protein concentrations in response to transcriptional changes. NAR also stabilises protein expression levels across populations of cells, correcting variations in transcription factor expression levels due to environmental noise [27]. These NAR pathways impact not only the cellular concentrations of self‐regulating transcription repressor proteins, but also the activity of downstream target genes that are regulated by these repressors [26].

Splicing factors within eukaryotic cells do not directly repress transcription from their own promoters. Instead, many splicing factors activate the splicing inclusion of PEs into their own mRNAs to introduce premature stop codons. However, the same conceptual advantages of NAR will apply. Changing either the transcription rate or level of PE splicing will lead to a different median splicing factor concentration at equilibrium. Following a change in splicing factor gene expression, NAR via PE splicing will then fine‐tune stable levels around the new median expression level, once the new threshold of splicing factor concentration is reached for efficient PE splicing. Importantly, NAR provided by PEs will enable rapid establishment of new splicing factor levels without over‐/under‐shooting, and respond to different levels of transcription.

For example, *TRA2B* transcription levels are controlled by transcription factors including MYC, so are upregulated in tumors that have increased MYC activity [28]. According to our hypothesis, NAR by the *TRA2B* PE would ensure a rapid and homogeneous response to MYC activation of *TRA2B*. The *TRA2B* PE contains multiple RNA‐binding sites for Tra2β protein (Figure 1A). As a result of increased *TRA2B* gene expression, there will be more *TRA2B* mRNA. Increased concentrations of Tra2β protein will then activate PE splicing inclusion within *TRA2B* mRNAs, thereby decreasing further production of protein‐coding *TRA2B* mRNAs, slowing down Tra2β protein production levels [29]. As a result of transcriptional induction by MYC of the *TRA2B* gene, NAR will ensure that a cell population will rapidly switch between expressing a lower median level of Tra2β protein to a homogeneous population of cells expressing a higher median concentration of Tra2β protein without overshooting to produce toxic protein levels (Figure 1C).

Maintaining proper expression levels of *TRA2B* is likely important since Tra2β protein regulates the splicing patterns of important genes in a concentration‐dependent manner [30]. Consistent with this, Tra2β expression is required for the development of the mouse embryo, brain, heart, and testis [31, 32, 33, 34]. Xenopus *Tra2b* is required for somite development [35]. Gene variants in the human *TRA2B* gene that cause production of a dominant negative version of Tra2β protein cause a neurodevelopmental syndrome that includes microcephaly and epilepsy [36].

## 3 PE Splicing Protects Meiotic Cells From Toxic Levels of Splicing Factor Expression

Our hypothesis presented here is based on analysis of a conditional animal mouse model that can identify specific tissues or cell types that depend on *Tra2b* PE splicing for function or survival in vivo [34]. Analysis of this mouse model identified a crucial role for the *Tra2b* PE during a specialised form of cell division called meiosis [34]. In this mouse model, the *Tra2b* PE was conditionally deleted from germ cells using a *Vasa‐Cre* transgene. Germ cells (the cells that go on to produce sperm and eggs) lacking the *Tra2b* PE survived and developed normally in the embryo, and proliferated through multiple mitotic divisions after birth (surviving about 12 postnatal days). However, germ cells then died during meiosis, causing male infertility by almost completely preventing sperm production.

The pattern of cell death during male meiosis showed that the *Tra2b* PE is essential for meiosis. Meiotic cells normally undergo rapid and safe transitions in splicing factor gene expression (including *Tra2b* [37]). Meiosis takes around 8 days in mice, during which time homologous chromosomes pair and exchange genetic information (meiotic prophase), followed by two sequential divisions to produce haploid gametes. Unlike mitosis, where transcription is turned off, gene expression during meiosis is both active and dynamically modulated. In particular, there is a big increase in global transcription during the pachytene stage of meiosis (part of meiotic prophase) involving changes in chromatin marks and super enhancer activation [38, 39, 40, 41, 42]. There is also increased expression of Tra2β protein during pachytene [34]. According to our hypothesis, the rapid increase in Tra2β concentrations during pachytene must be driven by strong levels of transcriptional activation of the *Tra2b* gene. We predict the PE within the *Tra2b* gene will be important for increased Tra2β expression to occur rapidly and homogeneously, yet not overshoot. This will enable a population of cells with low Tra2β expression to switch rapidly to a population of pachytene cells with high Tra2β expression. Tra2β concentrations may be upregulated during pachytene to deal with an increased general level of pre‐mRNAs being transcribed at this time, or to regulate specific alternative splicing patterns.

## 4 Why are Increased Levels of Tra2β Toxic During Meiosis?

Why might NAR mediated by the *Tra2b* PE ensure cell survival during meiosis? We predict that this is because the PE prevents pathological over‐expression of Tra2β in response to uncontrolled transcriptional induction. After conditional deletion of the *Tra2b* PE, the *Tra2b* gene would still be controlled by its endogenous promoter, and Tra2β will be strongly up‐regulated during pachytene. However, without its PE, Tra2β concentration levels will no longer be restrained by NAR and will be controlled only by the half‐lives of the *Tra2b* mRNA and Tra2β protein. According to our proposed hypothesis, this will increase both the median cellular expression of Tra2β, and the standard deviation of expression levels between different cells (Figure 1C).

Tra2β is a potent splicing activator protein [43]. Unusually high levels of Tra2β protein may change the splicing pattern of mRNAs that are critical for the survival or function of pachytene cells, or cause the selection of “cryptic” splicing events that inactivate the function of other mRNAs important for meiosis. As a specific example, deletion of the *Tra2b* PE in male germ cells caused increased levels of Tra2β protein in the testis and activation of a Tra2β‐dependent cryptic splice site in the *Ptbp2* gene, resulting in an unstable *Ptbp2* mRNA. *Ptbp2* encodes the key splicing factor protein PTBP2 essential for normal meiosis and sperm production [44]. Thus, pachytene cells might be vulnerable to loss of the *Tra2b* PE because increased Tra2β causes splicing errors affecting key proteins like PTBP2 that are needed for meiosis [34]. Alternatively, the production of aberrant spliced mRNAs caused by increased levels of Tra2β may also damage pachytene cells by creating double‐stranded RNAs that activate the innate immune response, or by creating mis‐spliced RNAs that form R loops [45, 46].

Interestingly, despite its key role in meiosis, the mitotic stages of germ cell development did not rely on the *Tra2b* PE for survival [34]. The mitotic cells did, however, require the *Tra2b* gene itself, since conditional inactivation of whole *Tra2b* gene function by deletion of an essential protein‐coding exon using the same *Vasa‐Cre* transgene caused death of mitotically proliferating germ cells in the postnatal testis. Hence, another insight from this new conditional mouse model [34] is that not all cells in the body that express Tra2β protein also depend on the *Tra2B* PE for function or survival, only particular cell types.

The complete male infertility caused by *Tra2b* PE deletion within the germline [34] helps explain its pattern of genomic ultra‐conservation. Sperm production is at the cutting edge of natural selection, since defects in sperm production would reduce genetic contribution to the next generation. Adult mice produce ∼150 million sperm/day, making it one of the most active developmental pathways operating in adults. The strong phenotypic effect caused by conditional deletion of the *Tra2b* PE is particularly striking compared to removal of other ultra‐conserved genome regions that have been implicated in controlling transcription (particularly as enhancer sequences). These latter studies detected only subtle phenotypic defects in mice and that did not reduce the ability to reproduce or survive within the laboratory environment [12].

Interestingly, the above mouse genetic analysis also showed that the *Tra2b* PE is critically important during other developmental pathways, for reasons that still have to be discovered [34]. Conditional germ‐line deletion of the *Tra2b* PE caused female infertility [34]. There was also evidence of a wider dependence on the *Tra2b* PE in normal development [34]. *Vasa‐Cre* is mainly active in the germline, but has some “leakiness” (meaning it can cause more general deletion in the body during development) [47]. Breeding analysis of *Tra2b* PE knock‐out mice revealed a 32% reduction in mice born with homozygous germ cell deletion of the *Tra2b* PE, compared to expectations of Mendelian genotype ratios [34]. This dramatic reduction in mice with homozygous deletion of the *Tra2b* PE is consistent with inappropriate deletion of the *Tra2b* PE outside the germline, caused by leaky *Vasa‐Cre* during early development, leading to embryonic lethality [34, 47]. Whether this general dependence on the *Tra2b* PE for normal development is for survival of key cell types or for ensuring normal cell function still needs to be established experimentally.

## 5 PEs Also Help Leverage Splicing Factor Expression During Development and Physiological Responses

This new hypothesis suggests that NAR by PEs operates in partnership with transcription to enable rapid changes in expression. How does this new hypothesis about splicing factor PE function fit into current understanding?

Thus far, the main focus of attention of PE function during development and pathology has been on the use of PE splicing as a controllable switch to increase/decrease expression of splicing factors. Recent analyses show that regulated *TRA2B* PE splicing inclusion controls Tra2β expression levels to induce mitotic cell proliferation of T cells. This is important for dealing with pathogens, and so likely also contributes to PE ultra‐conservation [48]. Before antigen stimulation, naïve populations of T cells circulate within the blood. Specific naïve T cell clones mitotically proliferate when antigens are presented to their T cell receptors by macrophages in complex with the MHC (Major Histocompatibility Complex), which activates a signalling response that controls *TRA2B* PE splicing and downstream levels of Tra2β protein expression. Tra2β protein levels within naïve T cells (before they are stimulated by antigen) are low because of high *TRA2B* PE splicing inclusion. Activation of the T cell receptor signalling pathway reduces *TRA2B* PE splicing inclusion, meaning more productive *TRA2B* mRNAs are made to raise Tra2β protein expression. The increased Tra2β protein levels then regulate mRNA splicing patterns important for increasing T cell numbers and driving cytokine secretion. Interestingly, this also shows that PEs can be incorporated into gene regulatory networks that are controlled by signalling pathways. The *TRA2B* PE splicing pattern switches again later in infection as antigen levels drop, with *TRA2B* PE splicing inclusion increasing as activated T cells switch to circulating memory T cells. Deletion of the *TRA2B* PE also prevents long‐term survival of memory T lymphocytes in the circulation [48].

Gene regulatory programmes in cancer cells often share similarities with developmental programmes. The operation of the *TRA2B* PE in controlling the proliferation of activated T cells is similar to the picture of PE function developed from studies in cultured cancer cells. CRISPR‐mediated deletion of ultra‐conserved PEs from splicing factor genes changed proliferation rates and cell survival in culture, predicting that ultra‐conserved PEs can both function as tumor suppressors and be important for cell viability [22, 49, 50]. CRISPR‐deletion of the *TRA2B* PE in human cancer cells increased both *TRA2B* expression and cell proliferation, indicating that normal intracellular levels of Tra2β protein are rate‐limiting for mitotic cell division [49]. Increases in *TRA2B* expression in human cancers can cause oncogenic patterns of splicing [22]. Using antisense oligonucleotides to increase *TRA2B* PE splicing inclusion has been proposed as a potential therapeutic approach to reduce Tra2β protein‐coding transcripts and tumor growth [22, 51]. As well as controlling mRNA stability, there is also evidence that splicing inclusion of the *TRA2B* PE causes expression of a long ncRNA that might act as a binding site for other RNA‐binding proteins [51, 52, 53]. More generally, there is also evidence from cultured cancer cells that PEs in some splicing factor genes (although not *TRA2B*) enable the selection of new reading frames within mRNAs rather than targeting them for NMD, leading to the expression of alternative splicing factor isoforms [54].

## 6 PEs may be Used as Versatile Motifs to Control Splicing Factor Expression

The current literature suggests, therefore, that individual splicing factor PEs are likely to operate within cell‐type‐specific gene regulatory networks. Although the models of PE function during germ cell development and T cell amplification are different, they have the similarity that in both cases, PE function is important when splicing factor gene expression levels are changing. Some features of *TRA2B* feedback control may also rely on interactions with other factors that are only expressed under certain circumstances. This can include signalling pathways (such as in T cells). PE splicing patterns can also be cross‐regulated by other splicing factors. For example, Tra2β protein also cross‐regulates a PE in the *TRA2A* gene that encodes a similar Tra2α protein, to control functional compensation from this homologue [22, 30].

Physiological differences in PE function across the body are again analogous to the negative autoregulation loops used by bacterial transcription factors, which are utilised as versatile recurring motifs within different gene expression networks [26]. The RBM3 splicing factor is important in neurons and provides another example of how PE splicing can be modulated in response to conditions. *RBM3* PE inclusion is regulated by the temperature‐controlled activity of another RNA‐binding protein called hnRNPH1. At reduced ambient temperature, there is increased binding of hnRNPH1 to the *RBM3* PE, resulting in increased PE splicing skipping and higher expression of RBM3 protein [55]. This is potentially clinically important. Reducing splicing inclusion of this *RBM3* PE using an antisense oligonucleotide increased expression of RBM3 protein, and provided some neuroprotection in a prion‐induced mouse model of neurodegeneration [55, 56]. More widely, temperature‐dependent splicing regulation of PEs has been implicated in controlling circadian rhythms [57]. Splicing factor gene PE inclusion also increases in cells exposed to hypoxia [50] and within differentiated smooth muscle cells [24], likely causing downstream global effects on gene expression patterns.

## 7 Conclusion

Recent investigations of splicing factor gene PE function in normal development and physiology have converged on the PE within the *TRA2B* gene [34, 48] (Figure 1A). Data from conditional knockout mice suggest the new hypothesis that this ultra‐conserved *Tra2b* PE enables rapid switches in Tra2β protein expression during meiotic prophase. The PE seems to be particularly important to protect these susceptible meiotic cell types from Tra2β over‐expression levels that interfere with normal cell function. However, other data from cancer cells and transplanted T cells point more to a role for the *TRA2B* PE in controlling mitotic proliferation. The observation that deletion of the same *TRA2B* PE can have different effects on the male germline (meiotic cell death) and on activated T cells (enhancing cell proliferation in response to cell signalling pathways but also reducing long‐term cell survival) indicates PEs are likely to have versatile and cell type‐specific roles in gene expression networks. Future analyses using other in vivo systems will establish the roles of the *TRA2B* PE in maintaining cell viability and in regulating the function and proliferation of cells. More widely, it is important to understand the fundamental functions of PEs before they can be safely therapeutically targeted [9].

## Author Contributions

D.J.E. and C.D. wrote the first draft of this article, and this was edited by A.J.M.W. and F.G.

## Conflicts of Interest

The authors have no conflicts of interest with this manuscript.

## Data Availability

Data sharing is not applicable to this article as no datasets were generated or analysed during the current study
